# Integrating hotspot dynamics and centers of diversity: a review of Indo-Australian Archipelago biogeographic evolution and conservation

**DOI:** 10.1007/s42995-025-00313-w

**Published:** 2025-07-30

**Authors:** Mingpan Huang, Michael J. Lawes, Wenliang Zhou, Fuwen Wei

**Affiliations:** 1 Center for Evolution and Conservation Biology, Southern Marine Science and Engineering Guangdong Laboratory (Guangzhou), Guangzhou, 511458 China; 2 School of Life Sciences, University of KwaZulu-Natal, Scottsville, 3209 South Africa; 3 Institute of Biodiversity and Environmental Conservation (IBEC), Universiti Malaysia Sarawak, 94300 Kota Samarahan, Sarawak Malaysia; 4 Jiangxi Key Laboratory of Conservation Biology, College of Forestry, Jiangxi Agricultural University, Nanchang, 330045 China; 5 CAS Key Laboratory of Animal Ecology and Conservation Biology, Institute of Zoology, Chinese Academy of Sciences, Beijing, 100101 China

**Keywords:** Longitudinal diversity pattern, Marine biodiversity hotspot, Dynamic centers hypothesis, Biogeography, Cryptic diversity, Multidimensional biodiversity conservation

## Abstract

The Indo-Australian Archipelago (IAA) is the world’s preeminent marine biodiversity hotspot, distinguished by its exceptional species richness in tropical shallow waters. This biodiversity has spurred extensive research into its evolutionary and biogeographic origins. Two prominent theoretical frameworks dominate explanations for the IAA’s biodiversity: the “centers-of hypotheses” and the “hopping hotspot hypothesis”. The “centers-of hypotheses” posits that specific regions serve as key sources of IAA biodiversity, either through the accumulation and overlap of species from external areas or via elevated rates of local speciation. In contrast, the “hopping hotspot hypothesis” asserts that biodiversity hotspots are dynamic, shifting across geological timescales in response to tectonic and environmental changes. This review synthesizes these contrasting perspectives into an integrated framework, the “Dynamic Centers Hypothesis,” which proposes that as biodiversity hotspots migrate over time, the IAA’s role in generating and sustaining biodiversity has evolved, with varying contributions from different sources dominating distinct historical phases. By synthesizing the evidence for both hypotheses and incorporating recent findings, including fossil and phylogeography data, we propose the “Dynamic Centers Hypothesis” as a comprehensive and unifying explanation for the IAA’s biodiversity. The review further explores biogeographic delineation, aligning tropical marine realms with the IAA’s evolutionary trajectory, from its Tethyan roots to its modern Indo-West Pacific dominance. Looking forward, advances in DNA barcoding and genomics are uncovering vast cryptic diversity, revolutionizing our comprehension of IAA phylogeographic history. These discoveries underscore the imperative for a multidimensional conservation framework, integrating phylogenetic, and functional diversity, to preserve this biodiversity hotspot amid escalating global change.

## Introduction

The oceans cover approximately 70% of the Earth’s surface and are widely regarded as the cradle of life. They support an extraordinary richness of marine biodiversity, encompassing 32 of the 34 animal phyla, with 14 of these endemic to the marine environment, including Ctenophora, Hemichordata, and Echinodermata (WoRMS Editorial Board 2024). The global biogeography of marine biodiversity has long fascinated ecologists. Recent integration of genetic data, functional traits, and big-data analytics has greatly advanced our understanding of these patterns. The Indo-Australian Archipelago (IAA) is widely recognized as the global marine biodiversity hotspot (Gagné et al. 2020; Hughes et al. 2002; Roberts et al. 2002; Tittensor et al. 2010; Yasuhara et al. 2022). While its precise boundaries remain under debate, most studies agree that this center encompasses Malaysia, the Philippines, Indonesia, and Papua New Guinea (Hoeksema 2007). The IAA exhibits a pronounced decline in species richness latitudinally toward the polar regions and longitudinally toward the eastern Pacific and western Indian Oceans, forming a distinctive “bull’s-eye” pattern, whereas a similar pattern and secondary biodiversity hotspot is observed in the Caribbean Sea in the western Atlantic ocean (Bellwood et al. 2012; Chaudhary et al. 2016; Tittensor et al. 2010). The bull’s-eye pattern is predominantly driven by shallow-water species, particularly reef-dependent organisms—such as corals, fishes, gastropods, and bivalves—which comprise a significant portion of marine biodiversity, leading to the IAA being commonly referred to as the Coral Triangle (Mora et al. 2003; Roberts et al. 2002; Tittensor et al. 2010; Veron et al. 2015).

The IAA’s bull’s-eye pattern underscores a distinct longitudinal diversity gradient, a phenomenon that is a persistent focus of marine biogeography. Unlike latitudinal diversity gradients (LDGs), which are often explained by factors such as temperature, seasonality, or productivity, longitudinal gradients typically defy these ecological explanations due to their minimal variation along these axes (Willig et al. 2003). Instead, they are more closely tied to historical processes operating over geological timescales, notably plate tectonics (Briggs 2007; Procheş et al. 2023; Renema et al. 2008; Tian et al. 2024; Yasuhara et al. 2017, 2022). Consequently, understanding marine biogeography and the formation of the IAA hotspot requires an approach that transcends contemporary ecological factors to include its historical drivers.

## Exploring the foundations: centers-of hypotheses

**Fig. 1:**
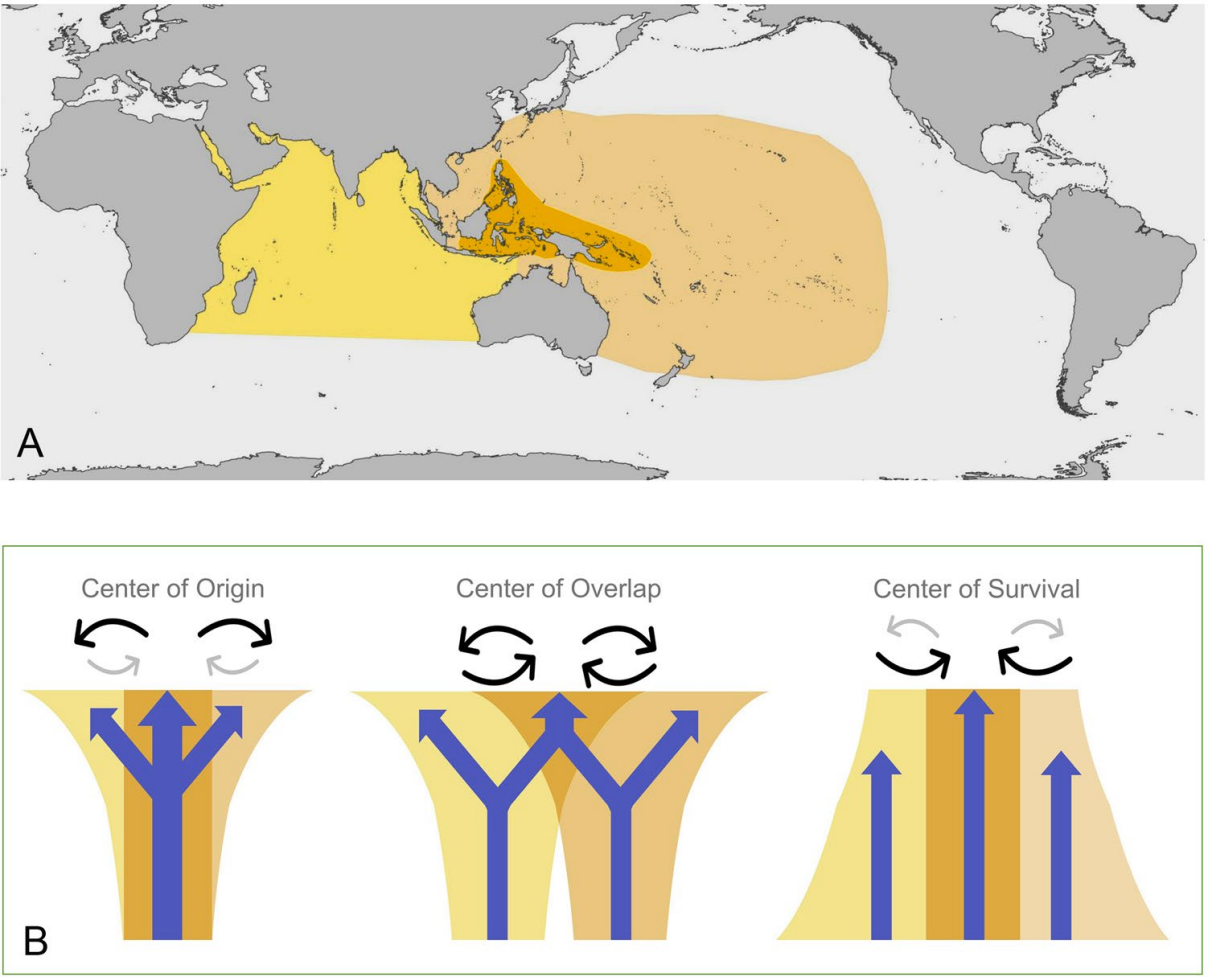
Map of the Indo-West Pacific regions (**A**) and concept models for the “Center of” hypotheses: **B** Center of Origin, **C** Center of Overlap, and **D** Center of Survival. The colors represent the geographical range and biota of the Indo-Australian Archipelago (Middle), Indian Ocean (Left), and West Pacific Ocean (Right) (Revised from Matias and Riginos, 2018)

In the twentieth century, various “centers-of hypotheses” have been proposed to explain the high biodiversity observed in the IAA (Palumbi 1997). These hypotheses emphasize distinct mechanisms that could account for the high concentration of species within the IAA, offering predictions about the locations and timing of species origins, as well as their patterns of range expansion or contraction (Bellwood et al. 2012; Cowman 2014) (Fig. 1). The “center of origin” hypothesis asserts that the IAA’s elevated biodiversity stems from an unusually high rate of speciation within the region, followed by the outward dispersal of newly formed species to adjacent areas (Briggs 1999a). Conversely, the “center of accumulation” hypothesis suggests that high biodiversity in the IAA is primarily due to the preferential colonization of that area by species originating elsewhere. This perspective highlights the influence of dispersal processes and biogeographic barriers, with ocean currents and historical shelf connections potentially facilitating species immigration into the archipelago, thereby enhancing its diversity (Bowen et al. 2013). The “center of overlap” hypothesis proposes that high biodiversity can arise in regions where the ranges of distinct biogeographic faunas converge and overlap. The IAA’s central location between the Indian and Pacific Oceans makes it a potential “center of overlap” for species from both regions (Barber et al. 2000). The mixing of species with different evolutionary histories and adaptations can lead to a significant increase in overall biodiversity within the zone of overlap. Finally, the “center of survival” hypothesis proposes that the IAA may have been a refuge for many marine shallow-water taxa, marked by a low extinction rate (Paulay 1990).

Testing these distinct hypotheses requires analysis of the geographic patterns of endemism and the relative ages of species or evolutionary lineages within and outside the IAA (Bellwood and Meyer 2009; Cowman et al. 2017; Reaka et al. 2008). Although the application of these approaches has stimulated decades of extensive research and debate (reviewed in Bellwood et al. 2012), there has been little consensus reached, underscoring the complexity of IAA biodiversity origins. However, in recent years, studies have increasingly emphasized the dynamic processes underlying the origin and maintenance of IAA biodiversity (Renema et al. 2008; Yasuhara et al. 2022). This shift has diminished debates over which “centers-of” hypothesis is most accurate, as focus has turned toward understanding the historical dynamics of hotspot formation.

## The hopping hotspot hypothesis: evidence for shifting centers of biodiversity

**Fig. 2:**
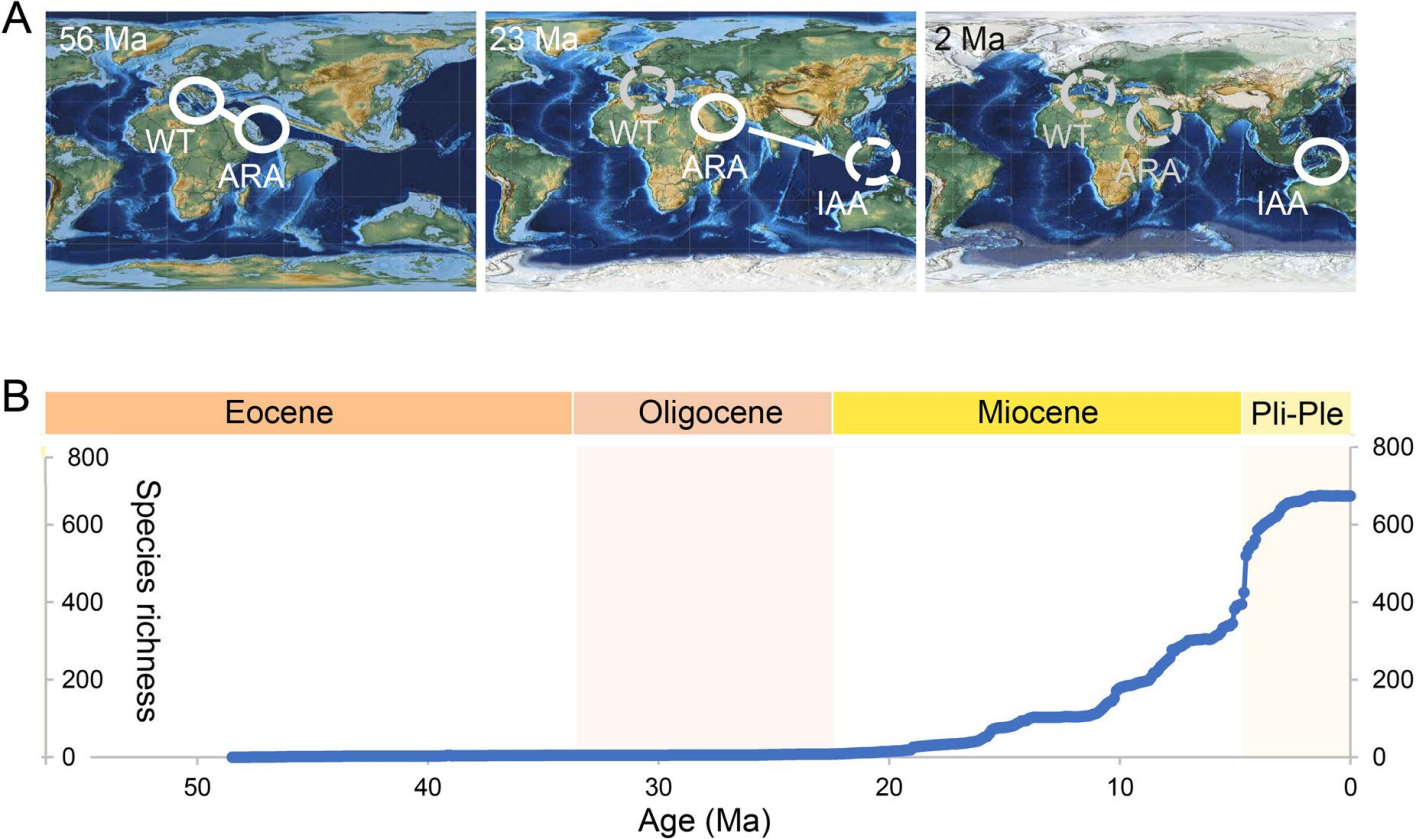
Hypothetical overview of Cenozoic global hopping hotspot dynamics and ostracod biodiversity trends in the IAA. **A** Arrows represent connectivity or dispersal pathways, circles indicate hotspot presence, and a dotted circle denotes vanishing biodiversity hotspots. WT, western Tethys hotspot; ARA, Arabian hotspot; IAA, Indo-Australian Archipelago hotspot. Adapted from Yasuhara et al. (2022). **B** Species richness of all ostracod fossils recorded in the IAA. Adapted from Tian et al. (2024)

The hopping hotspot hypothesis, initially articulated by Renema et al. (2008), introduces a dynamic view of biodiversity hotspots, suggesting that these areas of exceptional species richness experience spatial and temporal shifts driven by large-scale geological and environmental transformations. This dynamic view departs markedly from earlier, static models of biodiversity centers, suggesting that the locations of peak diversity are not fixed but rather migrate across vast regions over millions of years. This hypothesis outlines a potential migratory pathway for biodiversity hotspots, which suggests a westward origin in the Tethys Sea during the Eocene epoch (approximately 42–39 million years ago), a subsequent shift to the Arabian region by the late Miocene (around 20 million years ago), and a final relocation to the IAA by the Pleistocene epoch (approximately 1 million years ago) (Leprieur et al. 2016; Renema et al. 2008; Yasuhara et al. 2022) (Fig. 2A). These proposed movements are closely linked to significant geological events that reshaped marine habitats and influenced faunal dispersal. The closure of the Tethys Sea, a vast ancient ocean situated between the supercontinents of Gondwana and Laurasia, and the collision between the Australian and Southeast Asian tectonic plates are prime examples of such events (Gallagher et al. 2024; Hou and Li 2018; Wilson 2008). These tectonic activities dramatically altered ocean currents, created new shallow marine environments, and ultimately facilitated the migration and diversification of marine species (Yasuhara et al. 2022). Expanding on this narrative, recent research suggests the story may begin even earlier, providing evidence indicating that the western Atlantic may have acted as an earlier center of diversification for several key coral reef fish families (Cantalice et al. 2022). This finding suggests that the history of biodiversity hotspots may extend into the Paleogene, with an initial phase of diversification occurring in the western Atlantic prior to its eastward progression into the Tethys region.

The hopping hotspot hypothesis provides a compelling macro-scale framework, connecting biodiversity hotspots to the fundamental geological processes that shape Earth’s surface and oceans. By anchoring these shifts to major tectonic events, it provides a testable mechanism, enabling researchers to explore correlations between tectonic activity and the rise or decline of biodiversity centers. The supporting evidence for this eastward migration comes from the observed affinities between early Miocene Indian fauna and both older Tethyan (Eocene–Miocene) and younger Indo-Pacific (Miocene-Recent) assemblages (Harzhauser et al. 2009; Harzhauser and Fehse 2007; Renema 2007; Yasuhara et al. 2020a). This faunal continuity suggests a biogeographic linkage consistent with the eastward progression of a biodiversity hotspot, where species dispersed gradually from the Tethys, through the Arabian region, and into the emerging IAA (Harzhauser et al. 2009; Renema et al. 2008). Collectively, the hopping hotspot hypothesis synthesizes geological history with biodiversity patterns, deepening our insight into the mechanisms shaping the IAA’s remarkable species richness.

## Challenging the hopping hotspot model: the 'Whack-A-Mole' model—habitat suitability vs faunal tracking

In contrast to the ‘hopping hotspot’ model, which emphasizes the historical movement of specific faunal elements, the ‘whack-a-mole’ model presents an alternative perspective of the emergence of biodiversity hotspots (Yasuhara et al. 2022). This model serves as a null hypothesis, suggesting that biodiversity hotspots arise and fade in different locations over time, driven by in situ diversification spurred by favorable habitat conditions resulting from geological processes, rather than by the migration of faunal communities from earlier hotspots (Yasuhara et al. 2022). The core idea of the “whack-a-mole” model is that hotspots are like “moles” that pop up and go down independently in regions where environmental conditions support elevated diversity. The key factors such as habitat complexity, resource availability, and stable environmental conditions could lead to the independent emergence of high biodiversity in different regions at different times (Cowman and Bellwood 2011; Cowman et al. 2017; Mihaljević et al. 2017). This perspective diverges significantly from the “hopping hotspot' model’s focus on the eastward tracking of Tethyan faunal elements through successive geological periods. While the “hopping hotspot” model emphasizes the historical movement of lineages, the “whack-a-mole” model highlights the role of independently arising environmental suitability in shaping biodiversity patterns (Yasuhara et al. 2022). This dichotomy raises a fundamental question: to what degree does the IAA biodiversity result from the migration of species from earlier Tethyan or Arabian hotspots versus in situ speciation within the region? Research on cockles and giant clams (Bivalvia: Cardiidae) offers an example, suggesting that their diversity in the IAA arose from local lineages that diversified during the Miocene, rather than from Tethyan migration (Herrera et al. 2015). Resolving this issue necessitates a detailed examination of the relative contributions of external faunal migration—such as from Tethyan or peripheral archipelagos sources—and local diversification processes to the IAA’s biodiversity (Bowen et al. 2013; Cowman and Bellwood 2013a). While studies like those of Herrera et al. (2015) and Tian et al. (2024) provide partial insights, comprehensive analyses integrating phylogeny and fossils are still ongoing to fully disentangle these contributions. Such efforts are crucial for determining whether the IAA’s exceptional species richness reflects a legacy of historical dispersal or independent evolution driven by its unique ecological conditions, thus testing the predictions of these competing models.

## Integrating perspectives: the “Dynamic Centers Hypothesis”

The preceding discussion highlights that the “centers-of hypotheses” framework effectively categorizes potential roles for the IAA (e.g. origin, accumulation, survival, overlap) but tend to present these roles in a relatively static manner. By contrast, the “hopping hotspot hypothesis” effectively captures the dynamic historical shifts in biodiversity across geological time, tracing the movement of faunal elements through successive periods. While this model excels in outlining temporal and spatial transitions, it offers less clarity in distinguishing between local and external contributions to IAA biodiversity, particularly when confronted by the “whack-a-mole” model, which posits that hotspots arise independently through in situ diversification driven by favorable habitat conditions, rather than through the migration of faunal assemblages from earlier hotspots (Yasuhara et al. 2022). Notably, these hypotheses are not entirely independent, as they share fundamental processes like migration, speciation, and environmental adaptation, yet differ in their temporal and spatial emphases. To address these complementary strengths and limitations, this review builds on previous frameworks by integrating them into the “Dynamic Centers Hypothesis”. This synthesized hypothesis posits that the sources of IAA biodiversity—ranging from Tethyan migration and local speciation to peripheral inputs—vary across distinct phases of hotspot migration, with different “centers-of” mechanisms predominating at different historical junctures. As the IAA has undergone geographic shifts over geological time, propelled by abiotic factors such as tectonic movements and environmental changes, it has simultaneously experienced transitions in its functional roles as a generator and sustainer of biodiversity. The critical questions, then, are when and where these processes have been most influential, and how their interactions have shaped the biodiversity observed today. Specifically, this “Dynamic Centers Hypothesis” proposes the following temporal dynamics to elucidate these shifts:

(i) Eocene to the Oligocene: center of fauna accumulation

From the Eocene to the Oligocene, the accumulation of marine taxa migrating from the Tethys Ocean may have been a primary driver of the IAA’s burgeoning biodiversity. Tectonic changes of the Tethys Ocean, commencing in the early Cenozoic, played a critical role in establishing the IAA as a center of accumulation for shallow-water tropical marine species. The Tethys Ocean originated with the Triassic–Jurassic breakup of Pangea into Laurasia and Gondwana, forming a vast shallow ocean that fostered an early global tropical marine biodiversity hotspot (Zhu et al. 2022). This is evidenced by the diverse molluscan and ostracod assemblages, as well as the Monte Bolca reef fish fauna, which reflect a thriving ecosystem (Bellwood 1996; Friedman and Carnevale 2018; Harzhauser et al. 2002). Crucially, the Tethys Ocean established a circumtropical marine corridor, facilitating low-latitude water mass exchange among the Atlantic, Indian, and Pacific Oceans until the Neogene, a connectivity that proved essential for subsequent faunal migrations to the IAA (Hou and Li 2018).

The northward drift of the African and Indian plates during the Late Cretaceous (~ 80 Ma) initiated the gradual closure of the Tethys Ocean, reducing shallow-water habitats and prompting an eastward migration of species (Zhu et al. 2022). This process generated several distinct hotspots, such as the western Tethyan and Arabian hotspots, aligning with major tectonic events as described by the hopping hotspot hypothesis ( Leprieur et al. 2016; Renema et al. 2008; Yasuhara et al. 2022). Throughout this period, the IAA provided a persistent refuge with favorable habitats, sheltering incoming species and amassing considerable biodiversity. Fossil evidence indicates that the IAA gained significant diversity following the decline of the western Tethyan and Arabian hotspot, notably through some taxa of large benthic foraminifera, reef corals, and mollusks (bivalves and gastropods), all of which trace their ancestral origins to the Tethys Ocean (Renema 2007; Renema et al. 2008). Phylogeographic reconstructions of modern fish and invertebrate groups, including Holocentrids, Clupeoidei, calcareous tubeworm *Hydroides*, and Comatulids, further suggest that the IAA accumulated marine shallow water species originating from the Tethys Ocean during the Paleocene and Eocene (Dornburg et al. 2015; Lavoué et al. 2013, 2013; Saulsbury and Baumiller 2023). Thus, the tectonic evolution of the Tethys Ocean—from its role as an expansive circumtropical conduit to its eventual closure—underpinned the IAA’s emergence as a key accumulation center, blending migratory legacies with local ecological opportunities to establish the foundation of its remarkable reef fauna across geological time.

(ii) Miocene: center of origin

During the Miocene, in situ speciation emerged as the primary driver of biodiversity increase within the IAA. The collision of the Australian and Southeast Asian tectonic plates created extensive shallow-water and mosaic habitats, while concurrent climatic transitions likely accelerated the diversification of numerous coral reef-associated clades, encompassing both endemic lineages and those previously dispersed from hopping hotspots (Cowman and Bellwood 2011; Leprieur et al. 2016; Williams and Duda Jr 2008). In this period, newer, potentially more competitive species may have gradually radiated outward from the IAA, displacing older taxa to the periphery of their ranges (Briggs 1999b). This dynamic leads to several testable predictions: (1) species within the IAA should exhibit elevated in situ speciation rates over evolutionary time; (2) species near the IAA should be relatively younger, with peripheral regions serving as refuges for older taxa; and (3) the earliest fossil records of various taxonomic groups should predominantly originate within the IAA (Bellwood et al. 2012). Empirical support for these predictions comes from both phylogeny data and fossil records. Phylogenetic reconstructions indicate that, during the Miocene, several extant diverse lineages—such as reef fish, cockles and giant clams (Bivalvia: Cardiidae)—initially diversified within the IAA before exporting diversity to adjacent regions, including as far as the Caribbean Sea via dispersal through the Isthmus of Panama prior to its closure (Chen and Borsa 2020; Herrera et al. 2015; Thacker 2015). This rapid lineage diversification not only amplified taxonomic richness but also enriched the functional diversity that distinguishes modern IAA assemblages (Bellwood et al. 2017; Floeter et al. 2018; Mihaljević et al. 2017). Fossil evidence further corroborates this pattern, highlighting the Miocene as a pivotal period for the diversification of taxa such as *Acropora* corals, cowries, tonnoids, and ostracods in the IAA, with many genera and species first appearing during this epoch (Bellwood and Meyer 2009; Renema et al. 2008). Collectively, the proliferation and outward expansion of these lineages during the Miocene established the IAA as a center of origin, laying the groundwork for its present-day exceptional biodiversity (Cowman and Bellwood 2011, 2013a).

(iii) Pliocene to Pleistocene: Center of Survival, Overlap, and Species Accumulation

The Pliocene to Pleistocene period was marked by significant climatic cooling and associated glacial-interglacial oscillations, profoundly influencing the IAA’s biodiversity dynamics (Ravelo et al. 2004). The "center of survival" mechanism underscores the IAA’s role as a refuge, where stable habitats enabled species persistence during environmental fluctuations. For instance, the preservation of coral reef habitats during Quaternary glacial periods—despite widespread habitat loss—correlates strongly with extant fish richness, suggesting that these refugia within the IAA safeguarded reef fish diversity during sea-level fluctuations (Pellissier et al. 2014). This low extinction rate in the IAA contrasts sharply with the Caribbean Sea, where oceanographic changes coincided with the loss of numerous erect and free-living cheilostome bryozoan and coral species, contributing to the modern disparity in species richness between these regions (Budd et al. 2011; Di Martino et al. 2018).

Concurrently, Quaternary glacial oscillations drove the separation of ocean basins during Pleistocene eustatic cycles, promoting vicariance and speciation. Subsequent dispersal in the Holocene likely facilitated the coexistence of sister species, establishing the IAA as a center of species overlap (Bellwood et al. 2012). Although some studies interpret this pattern as evidence for the “center of origin” hypothesis, a key distinction exists. The overlap mechanism’s focus is on species diversification driven by basin vicariance and habitat mosaics during glacial cycles, and internal IAA overlap resulting from secondary contact during interglacial periods. This contrasts with the origin hypothesis’s sole focus on local speciation (Bellwood et al. 2012; Gaboriau et al. 2018; Tornabene et al. 2015). Phylogeographic studies reveal the convergence of sister species or intraspecific genetic differentiation near the shallow Sunda and Sahul shelves—spanning Indonesia, Malaysia, and northern Australia—potentially delineating a marine Wallace Line and supporting this overlap hypothesis (Barber et al. 2000; Bowen et al. 2016; Gaither et al. 2011; Gaither and Rocha 2013; Hsu et al. 2025; Puckridge et al. 2013; Sorenson et al. 2014). This pattern may be more prevalent among small taxonomic groups with restricted dispersal capabilities. For instance, cryptobenthic dwarf gobies (Gobiidae: *Eviota*) experienced rapid speciation during the Pleistocene, producing over 90 species (Tornabene et al. 2015). Allopatric speciation facilitated by transient or “soft” physical barriers during glacial cycles, or sympatric speciation driven by niche partitioning likely drove this process. Later, secondary contact may have enriched the IAA’s exceptional dwarf gobies diversity (Tornabene et al. 2015).

Finally, during this period, young species originating in peripheral habitats—such as the Hawaiian and Polynesian archipelagos—were able to disperse into the IAA via prevailing ocean currents, making the IAA a “center of species accumulation”, augmenting its exceptional diversity (Bowen et al. 2013; Evans et al. 2016). The genetic analyses demonstrate that these peripheral regions are not evolutionary dead-ends but rather sources of innovation, with neo-endemic reef fish emerging in the Pliocene at locations like Rapa Nui and Lord Howe Islands, potentially contributing to the IAA’s species pool (Budd and Pandolfi 2010; Delrieu-Trottin et al. 2019; Ma et al. 2018; Samayoa et al. 2022). Phylogeographic evidence further indicates that, while many wide-ranging Indo-Pacific species occur throughout the region, larval dispersal or distinct lineages from Hawaii also bolster IAA diversity (Bowen et al. 2013; Fitzpatrick et al. 2011). Together, these processes—survival, overlap, and accumulation—underscore the IAA’s complex biodiversity origins during the Pliocene and Pleistocene.

This Dynamic Centers framework elucidates the multifaceted origins of the IAA’s biodiversity, integrating diverse sources such as Tethyan migration (consistent with the hopping hotspot hypothesis), local speciation (center of origin), and contributions from the peripheral East Pacific islands and other adjacent regions (center of accumulation and overlap). The temporal variation in the prominence of these sources reflects a dynamic interplay between migratory and in situ processes, overcoming the limitations of earlier models by synthesizing these perspectives and acknowledging the contributions of multiple regions across extended geological timescales.

## Supporting evidence and new insights into IAA diversification

Recent high-resolution fossil data on ostracods from the IAA since the Cenozoic (Tian et al. 2024), provides a critical reference for elucidating the region’s diversification history, exemplifying the importance of an integrative perspective like the “Dynamic Centers Hypothesis”. This comprehensive dataset reveals a unidirectional diversification trend starting approximately 25 million years ago, characterized by a logistic rise in species richness that stabilized around 2.6 million years ago (Fig. 2B). During the Eocene to early Miocene, a marked increase in the diversity of Tethyan genera within the IAA points to substantial contributions from Tethyan migration, reinforcing the region’s role as a center of accumulation in this period (Tian et al. 2024). Following the Miocene, a decline in these Tethyan lineages coincides with the emergence of cosmopolitan and IAA-endemic species, signaling a faunal turnover and a transition from accumulation to origin centers, consistent with the “Dynamic Centers Hypothesis”. This shift is further supported by habitat availability, driven by tectonic collisions, and environmental improvements—such as reduced thermal stress after 13.9 million years ago—which facilitated in situ speciation (Hall et al. 2011; Renema et al. 2008; Tian et al. 2024; Westerhold et al. 2020; Wilson 2015). Expansive shallow marine habitats, resulting from these tectonic events, likely provided critical ecological niches for local diversification, not only within the IAA but also in transitional zones like the Arabian hotspot, echoing the “whack-a-mole” model’s emphasis on environmental suitability as a driver of diversity (Cowman and Bellwood 2011; Yasuhara et al. 2022). Furthermore, the absence of major extinction events in the IAA following the Pliocene, highlights the significance of center of survival during this period, solidifying its status as the world’s preeminent marine biodiversity hotspot (Tian et al. 2024).

In conclusion, the enduring biogeographic imprint of Tethyan ostracod taxa indicates that neither the hopping hotspot hypothesis nor the centers-of hypotheses alone can fully encapsulate the IAA’s intricate origins. This complexity underscores the value of an integrated framework like the “Dynamic Centers Hypothesis”, which accommodates the interplay of migratory legacies and local diversification across geological timescales. These insights pave the way for quantitative assessments of the IAA’s biodiversity sources, offering a refined understanding of its evolutionary trajectory.

## Biogeographic delineation and evolutionary dynamics of the IAA

**Fig. 3:**
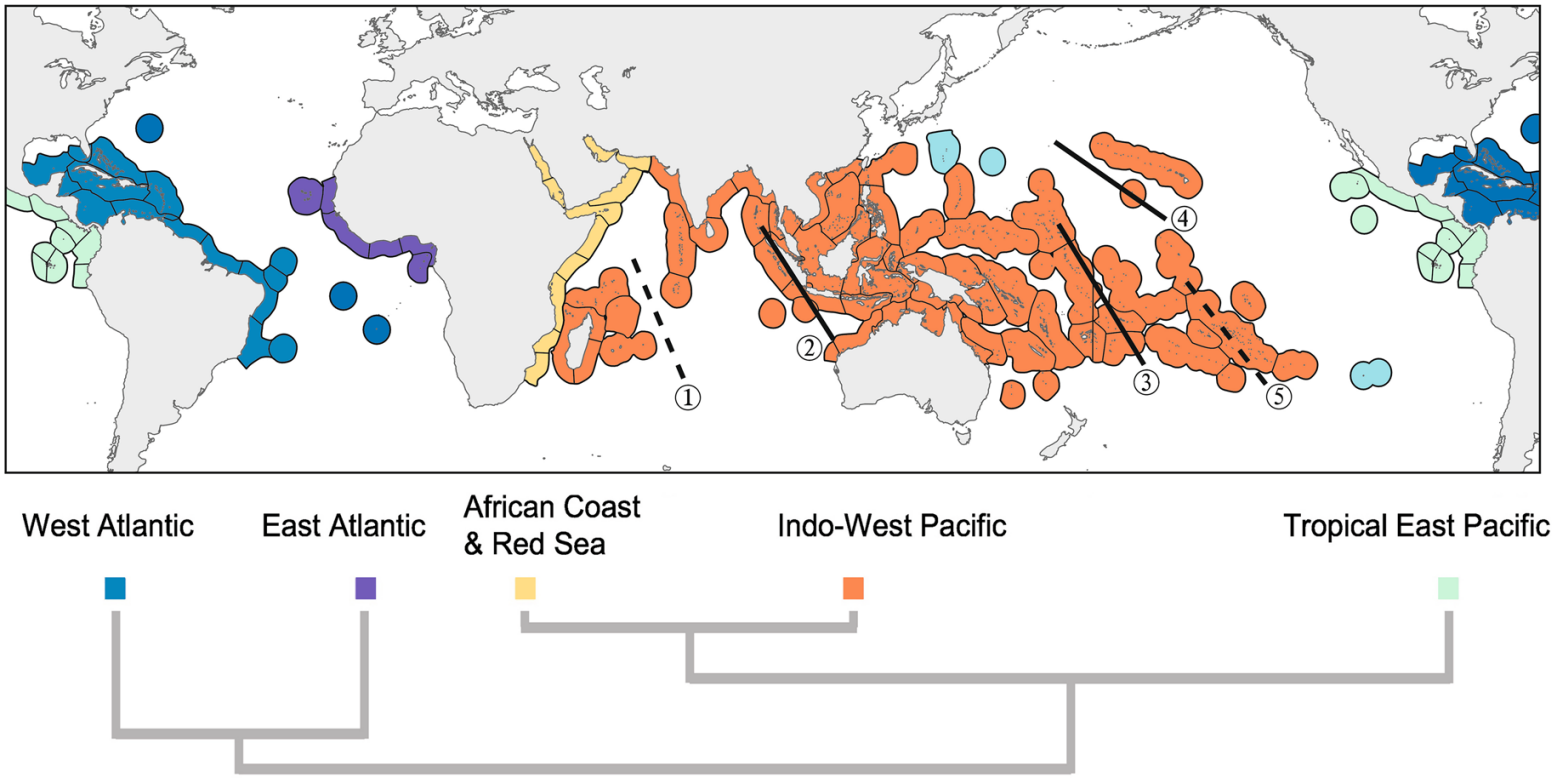
Biogeographic realms of extant tropical marine coastal faunas. The scheme and dendrogram are adapted from Cowman et al. (2017). Solid dark lines (②③④) indicate proposed barriers within Indo-West Pacific based on Crandall et al. (2019), Keith et al. (2013), Kulbicki et al. (2013), and Veron et al. (2015), with dashed lines (①⑤) indicating undetermined boundaries. Marine ecoregions follow Spalding et al. (2007)

Delineating biogeographic regions offers valuable insights for interpreting the evolutionary dynamics that have shaped the IAA and generated its distinctive “bull's-eye” biodiversity pattern. Early classification schemes prioritized taxonomic dissimilarity or endemism as a defining criterion (Briggs 1974; Ekman 1953; Keith et al. 2013; Kulbicki et al. 2013; Mouillot et al. 2013; Spalding et al. 2007), but contemporary approaches increasingly incorporate phylogenetic relationships to refine these regional distinctions (Cowman et al. 2017) (Fig. 3). Such analyses have illuminated the roles of oceanographic barriers—such as the Isthmus of Panama and historical events, including the Tethys closure and glacial cycles, in shaping IAA speciation and dispersal dynamics.

Cowman et al. (2017) integrates reef fish phylogenetic diversity with ancestral biogeographic reconstructions, identifying seven tropical marine realms: the Western and Eastern Atlantic, the Tropical East Pacific, the African Coast and Red Sea, remote Pacific Islands, and the Indo-West Pacific. Comparable patterns emerge from Veron’s analysis of zooxanthellate scleractinia corals, reinforcing the consistency of these patterns across taxa (Veron et al. 2015). This framework reveals a profound phylogenetic divergence between the modern Indo-West Pacific and the Atlantic realm, reflecting their current geographic isolation (Cowman et al. 2017; Veron et al. 2015). Historical reconstructions suggest that during the Oligocene (~ 27 Ma), the Caribbean stood as the most basal biogeographic region, consistent with the hypothesis that it may represent the oldest biodiversity hotspot, predating the Tethys hotspot (Cowman et al. 2017; Cantalice et al. 2022). By contrast, the Eastern Atlantic exhibits a stronger biogeographic affinity with the Indian Ocean coast of Africa, likely tracing back to an ancient connection via the Tethys Sea during this period—a link further supported by fossil evidence (Yasuhara et al. 2019). Following the closure of the Tethys Sea, the IAA emerged as a center of origin, with newly evolved species dispersing into the Western Indian Ocean. As a result, Africa’s biogeographic composition diverged from the Atlantic and aligned more closely with the Indo-West Pacific since approximately 18 Ma (Cowman et al. 2017; Obura 2012). Notably, the Tropical East Pacific has shown marked divergence from the Atlantic fauna since around 27 Ma, highlighting the critical role of the Panama Isthmus in separating Pacific and Atlantic faunal assemblages (Bacon et al. 2015; Cowman et al. 2017; Lima et al. 2020; Thacker 2017).

Within the Indo-West Pacific, a distinct meridional pattern still emerges, defined by five permeable barriers identified through faunal dissimilarity and intraspecific genetic data (Cowman and Bellwood 2013b; Crandall et al. 2019). For instance, a substantial yet permeable barrier between Polynesia and the Eastern Tropical Pacific (ETP), distinguished by vast oceanic expanses and a scarcity of shallow-water habitats, constrains biotic exchange (Wood et al. 2016). These soft barriers underscore the interconnectedness of the Indo-Pacific realm while accentuating the IAA’s central role in driving speciation and radiating diversity outward across this meridionally structured oceanscape. Together, these findings demonstrate that the IAA’s biogeographic significance arises from its dual heritage: an early linkage to Tethyan dispersal and its capacity to generate and distribute biodiversity within the Indo-Pacific, shaped by both historical legacies and regional dynamics.

## Future perspectives

(i) Cryptic diversity revealed by DNA barcoding and conservation

Despite considerable progress in marine biodiversity research, a significant fraction of marine species—estimated to range from half to three-quarters, including approximately 20% of marine fish—remains undescribed (Costello et al. 2012; Costello and Chaudhary 2017; Eschmeyer et al. 2010). Revealing this hidden diversity can advance our understanding of the IAA biodiversity, its evolutionary drivers, and inform effective conservation strategies (Grupstra et al. 2024; Hubert et al. 2012, 2017; Stanhope et al. 2023). This challenge extends beyond shallow waters to adjacent mesophotic coral ecosystems (MCEs), which thrive in deeper, low-light conditions and may harbor unique communities with high endemism, yet remain underexplored (Lesser et al. 2018; Pyle and Copus 2019). Similarly, deep-sea ecosystems, potentially connected to IAA margins, demand advanced tools to reveal their biodiversity (Iguchi et al. 2024). However, traditional morphological classification methods often prove inadequate for detecting hidden diversity. In shallow coral reefs, this inadequacy is evident among cryptic benthic fishes (Victor 2015). These species, typically characterized by elusive behaviors and small body sizes, represent a substantial yet frequently underestimated contribution to the overall species richness of coral reef ecosystems (Bessey et al. 2023; Brandl et al. 2018, 2019). Likewise, characterizing biodiversity in technically challenging MCEs and deep-sea environments requires methods beyond conventional visual surveys (Arango et al. 2019; Stefanni et al. 2022). In response, DNA metabarcoding and environmental DNA (eDNA) technologies have emerged as transformative tools, markedly improving our capacity to identify new biodiversity and map geographical hotspots from shallow reefs down through deeper zones (Deiner et al. 2017; Lu et al. 2024; Miya 2022).

DNA metabarcoding employs mitochondrial genes as internal species markers, revealing remarkable genetic diversity and overcoming the inaccuracies or limitations of morphological identification due to phenotypic plasticity or confusion (Sun et al. 2018). This technique has proven crucial in identifying cryptic species—those genetically distinct yet morphologically subtle—offering vital insights into unrecognized diversification histories and testing hypotheses about the IAA’s evolutionary origins (Jacobina et al. 2020; Mathon et al. 2022). For instance, Hubert et al. (2012) sampled 141 fish species across the IAA and found that 67 exhibited divergent lineages on either side, supporting the “center of overlap” hypothesis linked to Pliocene–Pleistocene glacial cycles and secondary contact. Furthermore, DNA barcoding has revealed extensive undescribed coral diversity, including “cryptic lineages” that exhibit ecological specialization and varying thermal tolerance, which is critical for coral resilience to heat stress, a pressing conservation concern under climate change (Grupstra et al. 2024; Richards et al. 2016). These findings highlight the conservation potential of cryptic diversity, identifying previously overlooked species and traits essential for ecosystem stability and restoration, thus guiding targeted strategies to protect the IAA’s biodiversity amid growing threats.

Likewise, eDNA analysis, which detects genetic traces in seawater, excels at identifying elusive and cryptic species, such as small-bodied benthic reef fishes, and supports rapid, site-specific diversity assessments, particularly in remote coral reef systems (Marwayana et al. 2022; Miya 2022). This approach is instrumental in uncovering previously unrecognized diversity hotspots. For example, Erpenbeck et al. (2024) applied DNA barcoding to shallow-water demosponges (Demospongiae) across the Indo-Pacific, revealing substantial cryptic diversity and proposing that sponge endemism and diversity hotspots are concentrated in the Red Sea rather than the traditionally recognized IAA core. In conclusion, these studies demonstrate how DNA barcoding exposes species diversity overlooked by conventional methods and refines our understanding of biodiversity hotspots’ scope and characteristics. This establishes a solid foundation for exploring novel diversity patterns and their underlying mechanisms, particularly among understudied taxonomic groups.

(ii) Genomic insights into IAA biodiversity dynamics and conservation

Recent advancements in genomics and high-throughput sequencing have revolutionized our ability to explore marine biodiversity patterns and phylogeographic histories. The population genomic analyses of geographically distinct populations enable researchers to accurately reconstruct species diversification, trace dispersal pathways, and assess genetic connectivity. By resolving high-resolution phylogenetic relationship, genomic data can clarify whether IAA species trace their ancestry to the ancient Tethys region or arose through local differentiation within the IAA, shedding light on the relative contributions of these regions as sources or sinks of IAA biodiversity. This approach provides quantitative insights into the origins and dispersal dynamics of IAA biodiversity, offering fresh opportunities to evaluate the “centers-of hypotheses.” A notable example is a whole-genome phylogeographic study of the seahorse genus (*Hippocampus*), which identified its origin within the IAA prior to the Miocene (18–23 Ma) (Li et al. 2021a; Qu et al. 2023). Subsequent diversification and global dispersal via the open Tethys Seaway produced an evolutionary lineage encompassing over 50 species, challenging the predictions of the “hopping hotspot” model and highlighting the IAA’s role as an early center of origin (Li et al. 2021a). Furthermore, the Pairwise Sequentially Markovian Coalescent (PSMC) analysis of whole-genome data can reconstruct historical population dynamics of species, tracing changes in population size and bottleneck events. This approach has demonstrated how glacial-interglacial cycles or geological events—like tectonic shifts and sea-level changes—shaped species’ evolutionary trajectories over time (Li et al. 2021b). More importantly, PSMC-inferred demographic histories allow differentiation between ancient population declines associated with paleoclimatic shifts and recent anthropogenic impacts, providing critical insights for conservation prioritization in threatened taxa like sharks (Stanhope et al. 2023).

(iii) Implications arising from the multidimensional biodiversity framework for IAA biodiversity conservation

Global marine biodiversity faces unprecedented pressures from anthropogenic activities and climate change, placing the IAA hotspot at risk of decline (Chaudhary et al. 2021; Costello et al. 2023; Huang et al. 2024; Ramírez et al. 2017; Tian et al. 2024; Yasuhara et al. 2020b). Refining conservation priorities to protect as many species as possible with limited resources is thus a critical challenge (Asaad et al. 2018). Traditional conservation strategies typically focus on taxonomic diversity—measured as species richness—prioritizing areas of high species abundance or flagship species, often neglecting ecosystem degradation caused by declines in functional roles. In contrast, the emerging multidimensional biodiversity framework extends beyond mere species counts to include genetic and phylogenetic diversity, which quantifies evolutionary history and uniqueness (Faith 1992), and functional diversity, which underpins ecological roles and ecosystem services (Bellwood et al. 2019). Therefore, this approach can better redefine global and regional conservation priorities, preserving both evolutionary heritage and ecosystem functionality (Cadotte et al. 2011; Huang et al. 2023; Tucker et al. 2019; Zhao et al. 2024). For instance, a recent study demonstrates that protecting approximately 22% of the ocean could conserve ~ 95% of known taxonomic, genetic, and phylogenetic diversity, lending support to the Kunming-Montreal Global Biodiversity Framework’s (GBF) 30 × 30 target (Fan et al. 2023).

Beyond species diversity, multidimensional attributes also aid in identifying conservation priorities enriched with phylogenetically and functionally distinct species, which often do not align with species richness hotspots (Kondratyeva et al. 2019; Violle et al. 2017). For instance, geographic hotspots of reef fish functional rarity are concentrated in the Tropical Eastern Pacific, diverging from the IAA’s species diversity peaks (Grenié et al. 2018). Consequently, effective marine conservation strategies should prioritize regions identified through multidimensional assessments that integrate taxonomic with phylogenetic and functional attributes. This approach enables a more comprehensive evaluation of biodiversity value, supporting scientifically robust, and impactful conservation measures to address the ongoing biodiversity crisis in the IAA and beyond.

## Concluding remarks

We have explored the global marine biodiversity 'bull's eye' pattern and its historical drivers. Marine species richness peaks in the IAA and declines towards the poles and peripheral regions, forming a distinctive “bull's eye” pattern. Historical tectonic events, such as the closure of the Tethys Ocean and the collision of tectonic plates, have shaped the IAA into a dynamic center of biodiversity, transitioning through various roles—from a center of faunal accumulation to a center of origin and, more recently, a center of survival, overlap and species accumulation. Biogeographic delineation further enhances our understanding of the historical forces that have shaped marine biodiversity patterns. These historical contingencies, combined with contemporary environmental factors, continue to influence the diversity gradients of marine species, centered on the IAA hotspot of marine biodiversity.

While historical factors have profoundly shaped marine biodiversity patterns, contemporary human activities have significantly altered key environmental drivers, such as coral reef extent and sea surface temperature, posing severe threats to marine biodiversity. We highlight the importance of cryptic diversity as a new frontier of marine biodiversity, and the role of DNA barcoding and genomics in uncovering cryptic diversity and IAA biodiversity origins and dynamics. The emerging role of multi-dimensional assessment of diversity for conservation, that protects not only species richness but also the evolutionary history and ecological functions of marine organisms, is essential for ensuring the health and resilience of ocean ecosystems.

## Data Availability

All data in this study can be downloaded from the links in this article. Other information are accessible from the main text and supplementary information.
